# Corrosion Behaviors of ZrSi Coating by Laser Cladding on Zr-4 Alloy in High-Temperature Steam

**DOI:** 10.3390/ma18184402

**Published:** 2025-09-21

**Authors:** Dongliang Jin, Changda Zhu, Xiqiang Ma, Zhengxian Di, Shizhong Wei

**Affiliations:** 1 Longmen Laboratory, Luoyang 471023, China; ziranheping@163.com (D.J.); zhuchangda157260@163.com (C.Z.); 2 National Joint Engineering Research Center for Abrasion Control and Molding of Metal Materials, Henan University of Science and Technology, Luoyang 471023, China; 3 Henan Key Laboratory of Special Protective Materials, Luoyang Institute of Science and Technology, Luoyang 471023, China

**Keywords:** ZrSi coating, ZrSi_2_ oxidation, self-healing, Zr-4 alloy, laser cladding

## Abstract

Si powder was deposited onto the surface of Zr-4 alloy via laser cladding to enhance its high-temperature oxidation resistance. The high-power laser radiation and rapid solidification lead to a reaction between Si and Zr, resulting in the formation of a microstructure consisting of lath-like ZrSi_2_ and Si-rich phases. The oxidation behavior of the laser-cladding ZrSi coating was evaluated at 1100–1300 °C in water steam. The weight gain follows a parabolic law, and the oxidation activation energy of the ZrSi coating is 182.7 kJ mol^−1^. The oxides produced by ZrSi_2_ oxidation are mainly ZrSiO_4_, ZrO_2_, and SiO_2_, and, under high-temperature conditions, the relative content of ZrSiO_4_ in the oxide decreases with increasing temperature. The oxidation of the ZrSi_2_ phase induces significant growth stresses, which are susceptible to causing cracks in the oxide, facilitating accelerated oxygen diffusion into the coating. However, the amorphous SiO_2_ formed at 1300 °C, which may be softened and fluidized to enable a self-healing effect, can heal the cracks to diminish oxygen permeation into the coating, improving its oxidation resistance. The oxidation resistance of the laser cladding ZrSi coating is better than that of the Zr-4 alloy.

## 1. Introduction

The Fukushima accident in 2011 exposed that the rapid oxidation of zirconium (Zr) alloy fuel cladding could release massive heat and explosive hydrogen in high-temperature water vapor during loss of coolant accidents (LOCA), such as design basis (DB) or beyond design basis (BDB) scenarios [1], presenting serious risks to light water reactor safety as well as to the public. It is essential to enhance the performance of Zr alloy under DB and DBD scenarios. Great efforts have been devoted so far to overcoming this drawback of Zr alloy fuel cladding. Although several tailored compositions have been produced [2], the shortcomings of Zr alloy as the main composition have not been completely overcome at high temperatures. In the pursuit of managing the weakness of Zr alloy, the materials, e.g., FeCrAl-ODS alloys [3] and chemical vapor infiltrated SiC tube [4], have been investigated to substitute the Zr alloy as fuel cladding. The higher neutron cross-section of the FeCrAl alloy than Zr alloy significantly decreases the economic benefits of thermal neutrons, and the joining of the SiC tube end with other alloys has not been satisfactory. Coating technologies for the surface protection of Zr alloys have been investigated extensively over the past decade [5], attracting much attention due to their potential early application in water-cooled reactors. The potential of several materials, e.g., ceramics [6,7,8], alloys, and metal matrix composites [6,9,10], has been extensively investigated for their application as oxidation-resistant coatings on Zr alloys.

Furthermore, the radiation resistance and physical stability of coating materials constitute critical criteria for their application in reactor environments. The TiN coating deposited by magnetic sputtering decomposed locally under high-dose Xe^+^ irradiation [11]. Although the CrN layer exhibited a considerable oxidation resistance compared to the metallic Cr coating at high temperature [12], even in an ambient atmosphere at 1160 °C [13], the monolayer CrN exhibited a tendency toward amorphization when subjected to irradiation at an ambient temperature [10]. The MAX phase materials, e.g., Ti_3_SiC_2_ and Ti_2_AlC, displayed less oxide mass gain than that of the Zr alloys, and the physical microstructure of Ti_2_AlC presented better stability under simulated irradiation by first-principle calculation [14], but the volume expansions of Ti_3_SiC_2_ and Ti_3_AlC_2_ were, respectively, 1.5% along the *c*-axis and 4% after irradiation, accompanied by a substantial strength decrease [15]. The Cr coating exhibited 0.66% microstructure expansion under 25 dpa exposure at 400 °C [16], demonstrating great irradiation resistance. For the FeCrAl coating, the formation of Al_2_O_3_ oxide provided it with robust oxidation resistance at high temperature [17], but the strong eutectic reaction between Fe and Zr at 928 °C constrained its application at elevated temperatures [18]. A few investigations have reported that the Cr coating proved significant resistance against oxidation in both pressured water and high-temperature steam conditions due to the relatively persistent and stable Cr_2_O_3_ protecting layer [19]. However, the interdiffusion products, e.g., brittle ZrCr_2_ and eutectic phase with low melting-point between Cr coating and Zr alloy, posed substantial risks to the ductility and integrity of the Cr-Zr cladding system [20]. Moreover, Liu et al. has revealed the oxidation rate of the non-coated Zr alloy was lower than that of the alloy sheltered by the magnetron-sputtered Cr coating above ~1350 °C, where the liquid phase from a eutectic reaction of Cr and Zr at 1370 °C may motivate the rapid diffusion of oxygen and metal elements, and thus be responsible for the surface rumpling and severe oxidation of the materials [21]. To enhance the elevated temperature properties of chromium coating, the CrAl coatings were co-deposited by utilizing Cr and Al targets, and the formation of alumina caused a decreased oxide mass gain in comparison to Cr coatings [22]. Nevertheless, it is beneficial to address the internal oxidation and optimize the aluminum content of the CrAl coatings.

Yeom et al. have studied the oxidation behaviors of the ZrSi_2_ compound and demonstrated its potential as an accident-tolerant material for protecting Zr alloy fuel cladding during DB and DBD scenarios [23]. ZrSi_2_ exhibits great thermal stability, and its transformed ZrSi phase has a higher melting point above 2000 °C. Furthermore, the oxides from the oxidation of ZrSi_2_, e.g., ZrO_2_ and the composite ZrO_2_-SiO_2_ (ZrSiO_4_), exhibit enhanced thermal stability. The dense and continuous SiO_2_ and ZrO_2_ film can hinder the diffusion of oxygen at high temperatures, leading to notable oxidation resistance for the Zr-Si coating by magnetic sputtering [24]. The addition of nitrogen to the Zr-Si compound has a considerable effect on the oxidation resistance, and the nucleation of SiO*_x_*N*_y_* at grain boundaries can prevent oxygen from migrating inward as the temperature rises [25], but the performances are not yet available for both isothermal and higher temperature oxidation.

The typical length-to-diameter ratio (~400) of Zr alloy cladding with a length of 4 m challenges the fabrication methods of its protective coating. As for the production of the coating, various approaches have been employed in previous research, e.g., thermal spraying [26], magnetic sputtering [27], multi-arc ion plating [28], micro-arc oxidation [29], and laser cladding [30]. The density of coatings produced by thermal spraying is low, with the presence of interconnected pores in the coatings. This porosity is harmful because it reduces the coating capacity to effectively resist the penetration of corrosive media. The density of coatings deposited by physical vapor deposition (PVD) exceeds that by thermal spraying. It should be noted that the deposition efficiency of multi-arc ion plating is a little higher compared with magnetic sputtering [6], yet significantly lower than that of thermal spraying. Nevertheless, coating on real Zr alloy cladding may be challenging owing to the limitations of PVD process stability. Currently, the commercial PVD facility with large capacity is so far unavailable to accommodate the Zr alloy cladding, and the self-developed equipment is extremely costly. Laser cladding can efficiently deposit thick and dense coatings with a well-bonded interface between the coating and substrate, and the associated equipment is readily accessible [31]. Furthermore, the process of laser cladding has been extensively studied in terms of parameter regulation [32,33]. The ZrSi coating, which was deposited through a coaxial feeding method of laser printing with Zr and Si powders, exhibited admirable oxidation resistance for a short time at high temperature [34], but the coating contained a high porosity, and the Zr alloy at the interface was significantly oxidized. In this work, the dense ZrSi coating was manufactured by laser cladding. The oxidation behaviors of the coating were investigated in high-temperature steam at 1100–1300 °C, so as to examine the oxide growth mechanism of the ZrSi coating for prospective application as a protective coating on fuel cladding.

## 2. Materials and Methods

The commercial Si powder was provided by Oerlikon Metco Co., Ltd. (Shanghai, China) with a purity of 99.9% and an average particle size of 45 μm. The dimension of the zirconium alloy substrate (Zr-4) was 10 cm × 10 cm × 4 cm, and the compositions of the Zr-4 alloy were 0.33% Fe, 1.25% Sn, 0.03% Cr, and Zr as the balance. Before the coating fabrication, the Zr-4 alloy substrate was ground and polished using silicon carbide grit paper up to a final grit size of 2500, then ultrasonically cleaned in acetone for 10 min. The coating deposition was performed using a laser-cladding experimental system, including a 3 kW fiber laser (ALL-In-Light, PRECITEC, Shanghai, China) and an industrial robot (KR20 R1810-2, KUKA, Shanghai, China). The laser spot was circular with a diameter of 6 mm. The molten pool protection was carried out using argon gas at a flow rate of 15 L/min to resist the material oxidation during deposition, and the powder feeding gas was also argon gas at a flow rate of 12 L/min. A few process parameters could affect the quality and features of the coating, such as the energy density (*E*) and overlap rate, etc. The energy density was determined by the laser power and scanning speed, and can be calculated as follows: *E* = *P*/*vD*, where *P*, *v,* and *D* were the laser power, scanning speed, and diameter of the laser spot, respectively. The relative process parameters comprised laser power 1200 W, scanning speed 5 mm/s, overlap rate 30%, powder feeding 10 g/min, and energy density 40 J/mm^2^.

The large samples were cut into square pieces with a dimension of 10 mm × 10 mm by an electric spark cutting machine. Before coating oxidation, the coating surface was mechanically ground and polished, and then the specimens were ultrasonically cleaned in ethanol and acetone for 10 min. After polishing, the thickness of the laser cladding coating was 0.5 mm. The high-temperature oxidation in steam was conducted using a vacuum box furnace (volume: 3.4 L) to allow easy insertion and removal of specimens, which helped to avoid excessive oxidation of the materials during the furnace temperature ramp-up and cool-down processes. The deionized water steam was generated by an ultrasonic atomizer with a power level of 20 W and an atomization capacity of 500 mL/h. The water steam (5 g/L, 25 °C) was fed into the furnace using high-purity argon gas (99.999%) at a continuous flow rate of 40 sccm. The oxidation experiments were conducted at 1100–1300 °C for different times. As the furnace temperature reached the set value, the specimens were rapidly put into the furnace, and the evacuation process was completed within 45 s. Five specimens for each oxidation condition were exposed to minimize the error of mass gain. After oxidation, the specimen was embedded in transparent epoxy resin and ground to a final 3000 grit with silicon carbide grit paper and mechanically polished using 50 nm colloidal silica.

The optical cross-sectional image was obtained using a 3D measuring laser microscope (LEXT OLS5100, OLYMPUS, Nanjing, China). X-ray diffraction (XRD) measurement of the sample was performed by a diffractometer (Bruker, Beijing, China) with Cu Kα radiation at a scanning speed of 5°/min. The microstructure of the cross-section and surface morphology were observed using a scanning electron microscope (SEM, JSM-IT800, JEOL, Shanghai, China) coupled with an energy dispersive X-ray spectrometer (EDX, Oxford, UK). The surface chemistry of the specimens was analyzed by X-ray photoelectron spectroscopy (XPS, AXIS UltraDLD spectrometer from Kratos Analytical, Shanghai, China), whose excitation source utilized 1486.6 eV Al Kα radiation with a pass energy of 50 eV and a step size of 0.1 eV. XPS binding energy data were calibrated by employing C 1s photoelectron line (284.8 eV). Raman spectroscopy analysis for the surface oxides was carried out utilizing a LabRAMHR Evolution spectrometer (Horiba Jobin Yvon, Shanghai, China) equipped with a 532 nm Ar laser. The mass gain of the sample after steam oxidation was measured by a microbalance (METTLER TOLEDO, Shanghai, China) with an accuracy of 0.01 mg.

## 3. Results

### 3.1. As-Deposited Coating

**Figure 1:**
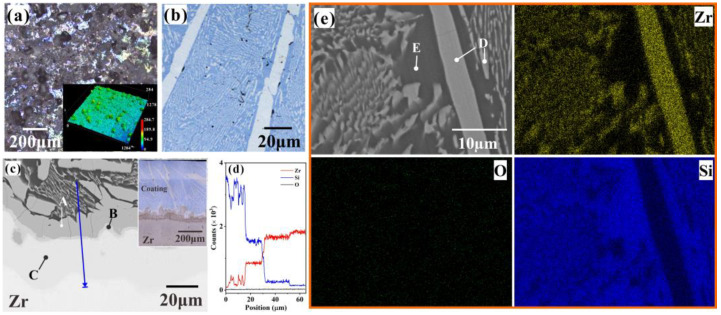
(**a**) Surface of the laser cladding ZrSi coating, and the inset is the 3D morphology of the coating. (**b**) The surface morphology of the ZrSi coating after mechanical polishing. (**c**) The cross-sectional image of the ZrSi coating, and the (**d**) elemental profile along the line in Figure 1c by EDS line scanning. (**e**) EDS element mapping of the cross-section of the laser cladding ZrSi coating.

After the coating deposition by laser cladding, the surface macrostructure of the coating is shown in Figure 1a, where the inset is a 3D morphology of the coating, and the surface roughness (Sa) is 8.245 µm. A large number of bulge particles can be observed on the surface, which is formed by the uneven disturbance in the molten pool that rapidly solidifies under a high-temperature gradient. Before heat exposure in steam, the coating surface is mechanically ground and polished, the surface roughness is Sa 0.028 µm, and its typical optical image is shown in Figure 1b, where the blue area is full of a white layered structure with a thickness of 2–5 µm and a length of ~50 µm. To further investigate the microstructure of the coating, its cross-sectional image is shown in Figure 1c. The coating contains many bright laths embedded in a blue matrix, where the length of the laths is about dozens to hundreds of micrometers. Combined with the morphology in Figure 1b, the microstructures of the laths are generally lath-like. The porosity of the coating is about 0.1% from the image analysis of the cross-sectional SEM images. Figure 1d displays the elemental profile along the blue line in Figure 1c by EDS. The interface between the coating and substrate is well bonded, and there are a few cracks that are mostly perpendicular to the interface. The interdiffusion zone mainly consists of three layers, which are marked by A, B, and C in Figure 1c, and the main compositions of the layers are listed in Table 1. The phase in the areas marked by A approximates to ZrSi_2_.

During the laser cladding of Si powder, the heat energy supplied by the laser melted the silicon powder and the outer layer of the Zr substrate, and the convection effect of the laser beam facilitated the mixing of materials within the molten pool [35], leading to the formation of new phases aligned with the temperature gradient. According to the results in Table 1, the temperature gradient is the highest close to the boundary between the coating and Zr-4 substrate, where it has a large quantity of ZrSi_2_ laths that even merge with one another to form a continuous layer labeled as A in Figure 1c. To further explore the content of the coating, the element mapping of the cross-section was performed by EDS (Figure 1e). The compositions of the areas labeled as D and E in Figure 1e are shown in Table 1. Being similar to the morphology in Figure 1b, the microstructures in the coating mainly consist of two kinds of laths, the large-scale lath with a length from dozens to hundreds of micrometers, and the small parallel-aligned lath. The large-scale lath is composed of Zr and Si, the stoichiometric ratio is about 1:2, and the ZrSi_2_ laths are dispersed in the Si matrix. The variation in lath size can be ascribed to the insufficient and inconsistent agitation of the molten pool and mass transfer during laser cladding procedures [36].

### 3.2. Oxidation of ZrSi Coating

**Figure 2:**
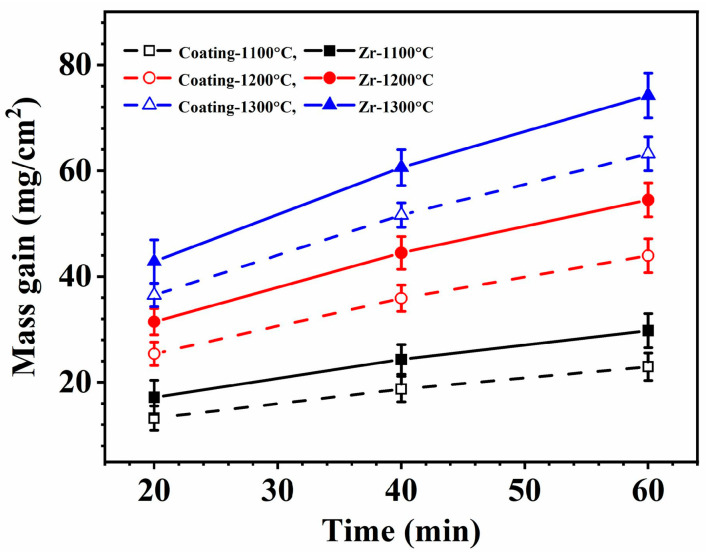
Relationship between the mass gains of the laser cladding ZrSi coating and Zr-4 alloy after oxidation and time in high-temperature steam conditions.

The protective effect of laser cladding ZrSi coatings on the oxidation resistance of Zr alloys was investigated at 1100–1300 °C in water–steam conditions. Figure 2 shows the relationship between the mass gain of the samples after oxidation and time under high-temperature water–steam conditions. It can be seen that the mass gain gradually increases over time; at the same temperature and time, the mass gain of the cladded coatings is less than that of the Zr-4. As the temperature rises, the mass weight-gain difference between Zr-4 and the coating gradually increases, such as the difference in mass gain at 1100 °C for 60 min is 6.8 mg/cm^2^, and the difference is 10.0 mg/cm^2^ at 1300 °C for 60 min. This indicates that the laser cladding ZrSi coating can provide oxidation protection for the Zr-4 alloy substrate, and the protective effect of the ZrSi coating is better at higher temperatures.

**Figure 3:**
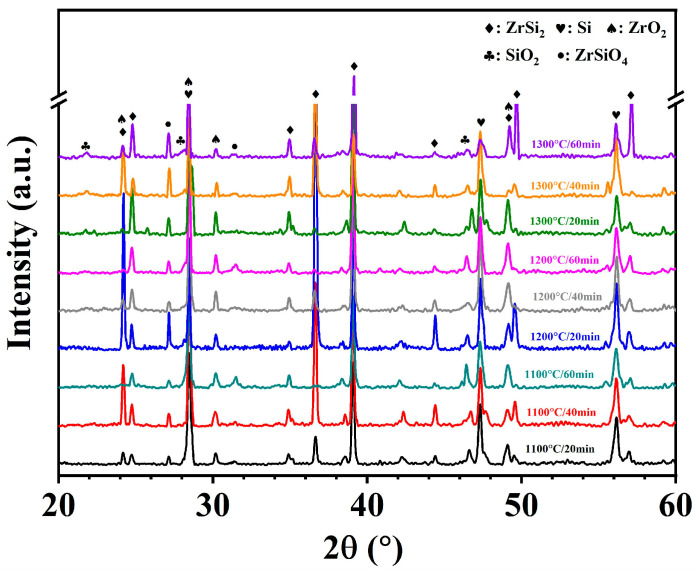
XRD patterns of the laser cladding ZrSi coatings after oxidation under different conditions.

**Figure 4:**
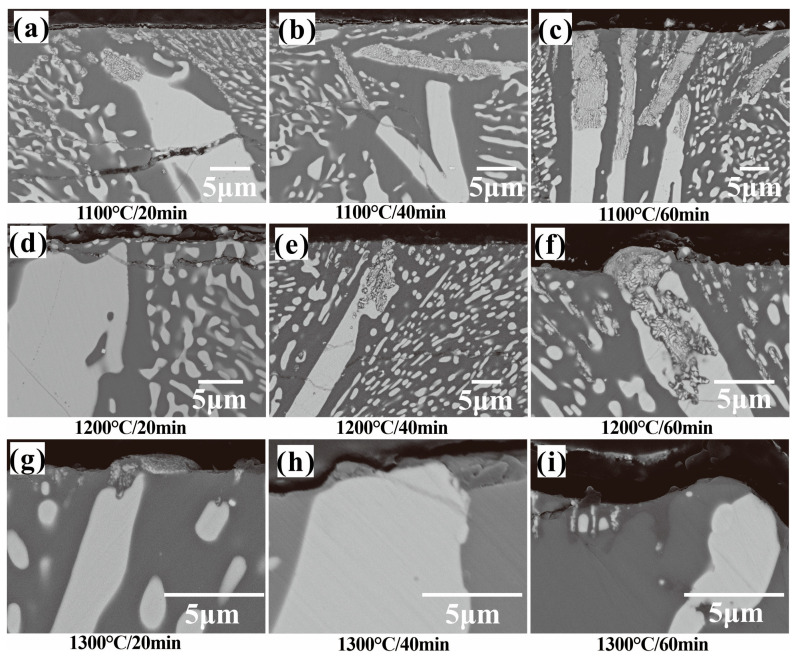
Microstructures of the cross-sections for the laser cladding ZrSi coating after oxidation, (**a**–**c**) 1100 °C, (**d**–**f**) 1200 °C, and (**g**–**i**) 1300 °C with oxidation time from 20 min to 60 min, respectively.

To further explore the oxidation behavior of laser cladding ZrSi coatings, it is necessary to investigate the compositions of the oxide layer formed on the ZrSi coating after high-temperature oxidation. The phases of the oxide layer were tested by XRD, as shown in Figure 3. After oxidation at 1100 °C for 20 min, the phases of the coating consist of ZrSi_2_ and Si [37], and the oxides are mainly ZrO_2_, ZrSiO_4_, and a small amount of SiO_2_. This is also consistent with the results shown in Table 1, in which the main components are ZrSi_2_ and Si for the laser cladding ZrSi coating. At 1100 °C, as the oxidation time increases to 60 min, the intensity of the diffraction peak of ZrSiO_4_ at 31.5° significantly increases, suggesting that the content of ZrSiO_4_ in the oxide layer gradually increases. As the temperature increases, especially at 1300 °C, SiO_2_ shows an obvious diffraction peak at 22°, indicating that the content of SiO_2_ increases significantly. In addition, there are always strong ZrSi_2_ diffraction peaks at 36.5° and 39.1°, and a strong Si diffraction peak at 28.4° after oxidation under different conditions. The energy of Cu Kα X-rays is ~8 keV for the XRD analysis, and its penetration depth is more than 5 μm with a relative intensity greater than 60% after penetration in high-density materials [38,39]. The thickness of the oxide layers formed on the coating is about 1–20 μm after oxidation, as shown in Figure 4. It can be inferred that X-rays can penetrate the oxide layer, thereby obtaining the phase diffractions of both the oxide layer and the ZrSi coating.

**Figure 5:**
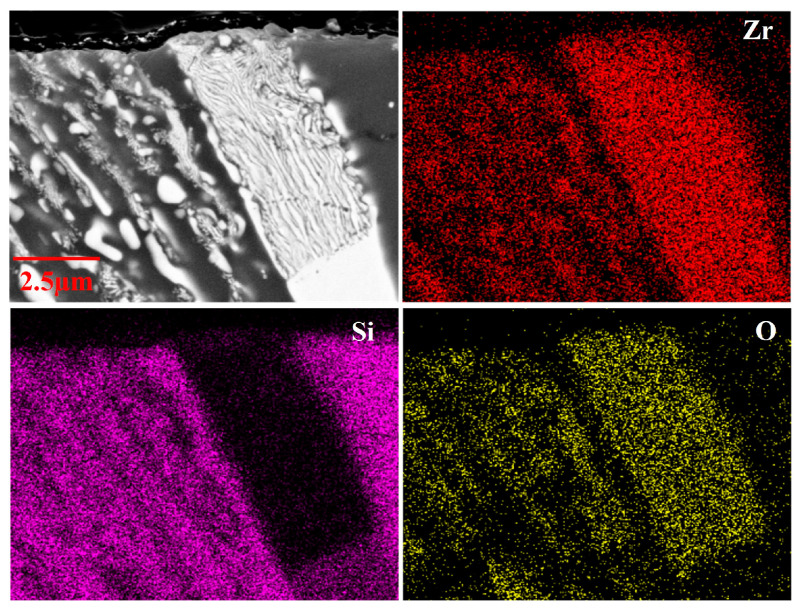
EDS mapping of the oxidized ZrSi coating at 1100 °C for 20 min.

From the cross-sectional images of the oxide layers of the laser cladding ZrSi coating after oxidation in Figure 4, it can be observed that the oxide thickness gradually increases over time at the same temperature. For the samples oxidized at 1100 °C in Figure 4a–c, the lath-like and granular ZrSi_2_ phases near the coating surface obviously break into lots of small pieces. To further study the oxide compositions, element mapping analysis was performed for the oxide layer, as shown in Figure 5. It can be seen that oxygen is mainly distributed in the oxides from ZrSi_2_ phases, while the oxygen in the Si phase is lower, and no uniform oxide layer is formed on the surface. The oxide layer of the Si phase might be pretty thin and could be damaged during the mechanical grinding and polishing process, making it difficult to detect using EDS analysis. The lower oxygen distribution in Si indicates that the diffusion rate of oxygen in the ZrSi_2_ phase is faster. The oxidation of ZrSi_2_ could generate ZrO_2_, ZrSiO_4_, and SiO_2_, leading to large lattice deformation of ZrSi_2_, thus producing large oxide growth stress, which can cause microcracks in the ZrSi_2_ phase and further cause its fragmentation. These microcracks can provide diffusion channels for oxygen diffusion, accelerating the oxidation rate of the ZrSi_2_ phase.

**Figure 6:**
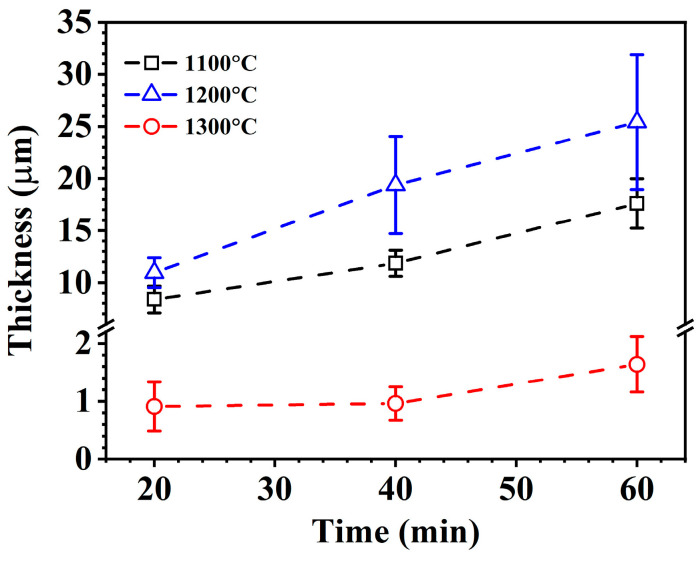
Average oxide thickness on large ZrSi_2_ laths vs. oxidation time.

**Figure 7:**
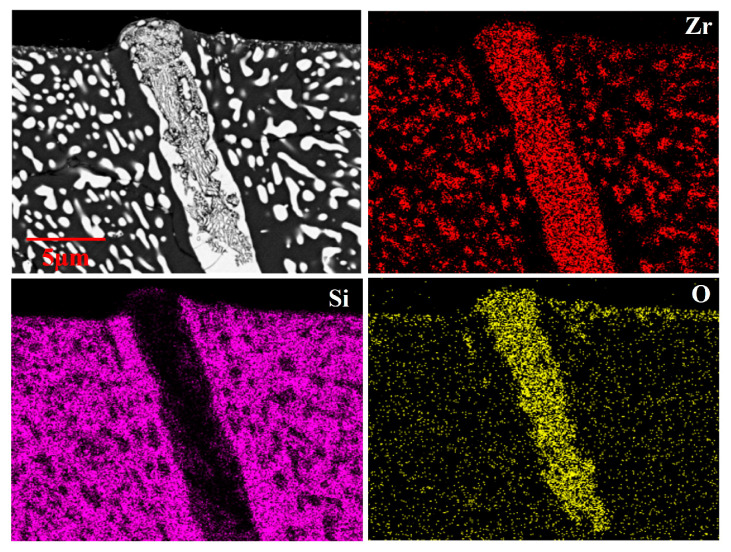
EDS element mapping of the ZrSi coating oxidized at 1200 °C for 60 min.

The cross-sectional microstructure of the oxidized ZrSi coating at 1200 °C is shown in Figure 4d–f. The average oxide thickness of ZrSi_2_ oxidized at 1200 °C is higher than that obtained at 1100 °C, which is derived from a Gaussian normal distribution of no fewer than 50 data points obtained from measurements of different SEM images for the oxidized large-scale ZrSi_2_ laths, as shown in Figure 6. Figure 7 shows the cross-sectional element mapping of the ZrSi coating oxidized at 1200 °C for 60 min. Compared with the microstructure obtained at 1100 °C, the oxides also display strip-like fragments for the large-scale lath-like ZrSi_2_ phase; however, the small-sized ZrSi_2_ granular phase below the coating surface does not undergo severe oxidation, and a continuous oxide layer is formed on the surface.

**Figure 8:**
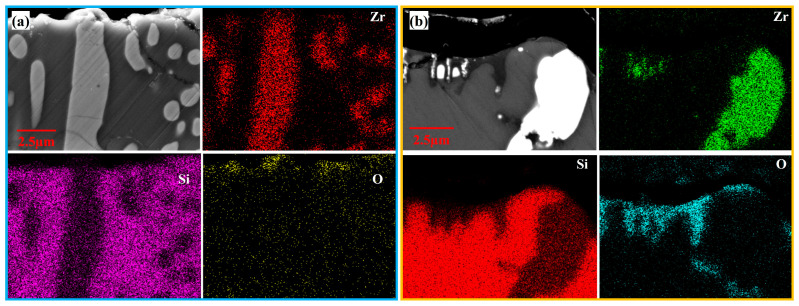
EDS elemental mapping of the ZrSi coating oxidized at 1300 °C for a time: (**a**) 20 min; and (**b**) 60 min.

The cross-sectional microstructure of the laser cladding ZrSi coating heated at 1300 °C is shown in Figure 4g–i. Compared with the microstructures obtained at lower temperatures, the small-sized ZrSi_2_ laths exhibit obvious spheroidization. As the temperature rises, the small ZrSi_2_ laths gradually spheroidize, and the sphericity is the highest under the condition of 1300 °C/60 min. After heat exposure at 1300 °C, the large ZrSi_2_ laths exhibit lesser oxidation than those exposed at a lower temperature, and the average oxide thickness of the large ZrSi_2_ laths is ~1.7 μm after oxidation for 60 min in Figure 6, which is much smaller than that formed at lower temperatures. Figure 8 shows the EDS elemental mapping of the ZrSi coating oxidized at 1300 °C. It can be seen from Figure 8a that the oxygen diffusion distance in the large ZrSi_2_ laths is relatively small, and a thin continuous oxide layer is formed on the coating surface. As the oxidation time is up to 60 min, the thickness of the oxide layer also increases, as shown in Figure 8b, and the small-sized ZrSi_2_ granular phase near the surface is also oxidized.

### 3.3. Oxide Surface Microstructure

**Figure 9:**
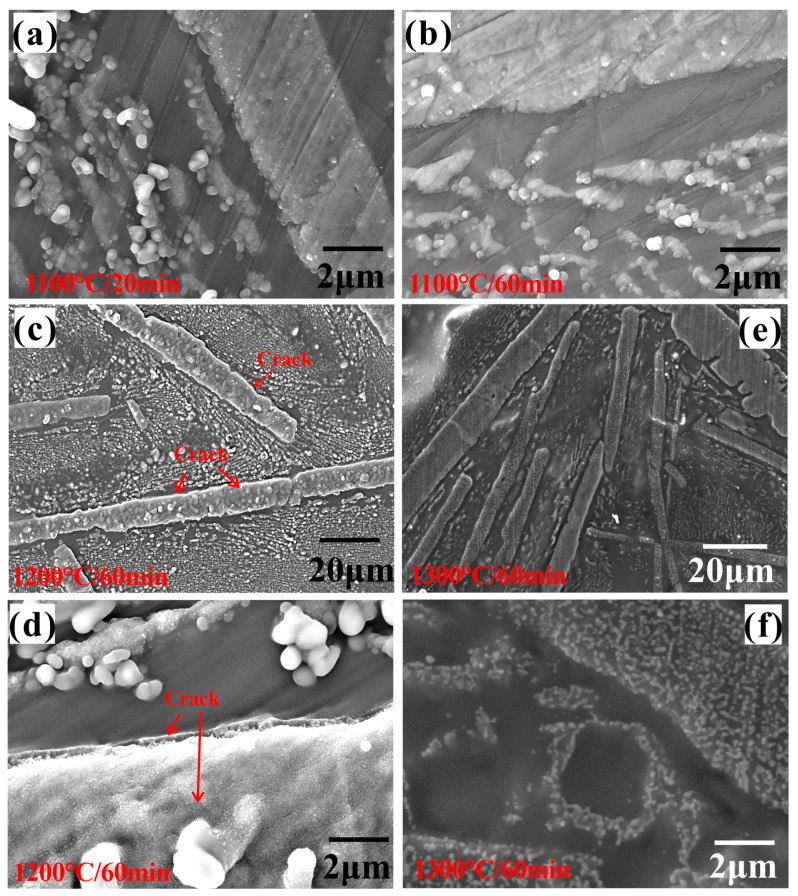
Surface morphologies of the oxidized ZrSi coating under different conditions: (**a**) 1100 °C/20 min, (**b**) 1100 °C/60 min, (**c**,**d**) 1200 °C/60 min, and (**e**,**f**) 1300 °C/60 min.

**Figure 10:**
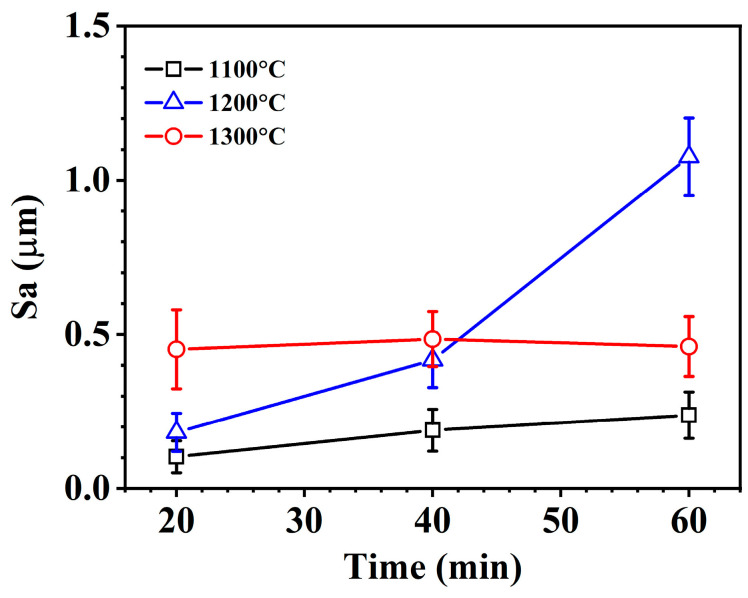
Surface roughness of the ZrSi coating heated at 1100–1300 °C for different times.

Figure 9 shows the surface microstructure of the oxidized ZrSi coating. Before oxidation in high-temperature steam, the surface roughness of the polished coating is Sa 0.028 µm. For the ZrSi coating oxidized at 1100 °C for 20 min (Figure 9a), the oxides of the large ZrSi_2_ lath show volume expansion and protrude from the surface, and the oxides from the small-sized ZrSi_2_ are granular; after oxidation for 60 min, no cracks appeared between the oxides and the Si phase around the lath-like ZrSi_2_, as shown in Figure 9b. After coating oxidation at 1200 °C for 60 min, cracks appeared within the boundary between the oxides from the large ZrSi_2_ lath and the surface (Figure 9c,d), which indicates that a large oxide growth stress occurred. For the ZrSi coating exposed at 1300 °C for 60 min, no large-scale oxide particles emerge on the surface, and many nano-sized oxide particles are formed in the oxide of the ZrSi_2_ lath, and no cracks appear in the oxide layer, as shown in Figure 9e,f. The crack density (*ρ*) of the oxide surface was quantitatively evaluated by averaging it from five SEM images, where its expression was *ρ* = Σ*L*/*A*, and Σ*L* was the sum of crack length in the image, and *A* was the area of the image. The crack densities were 1.7 ± 0.8 × 10^−2^ μm^−1^ and 8.1 ± 1.2 × 10^−4^ μm^−1^ for the oxide formed at 1200 °C for 60 min and 1300 °C for 60 min, respectively. To further analyze the changes in the coating surface morphology after oxidation, the surface roughness was measured, as shown in Figure 10. It can be seen that the surface roughness gradually increases over time as the temperature increases from 1100 °C to 1200 °C. The surface roughness of the coating oxide is relatively high after oxidation at 1300 °C for 20 min; however, it remains around 0.46 μm over time.

## 4. Discussion

### 4.1. ZrSi Coating Oxidation

**Figure 11:**
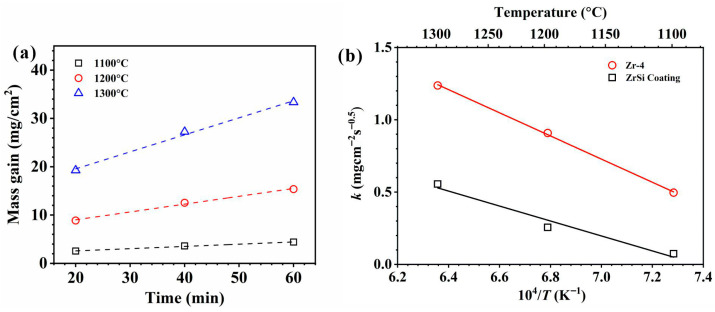
(**a**) Calculated weight gain of the laser cladding ZrSi coating within different conditions; and (**b**) Arrhenius plots of the diffusion coefficients for the ZrSi coating and Zr-4 alloy.

The laser cladding ZrSi coatings were exposed to high-temperature water–steam conditions. The obtained oxides are mainly ZrO_2_, ZrSiO_4_, and SiO_2_ (Figure 3), and the oxide mass gain gradually increases over time (as shown in Figure 3). The oxidation rate of a material relates to its composition, the real area within its geometric shape, temperature, gas composition, and the cyclic details of the oxidation process [40]. In this study, the heat exposure of the specimens is isothermal oxidation. The method of studying isothermal oxidation kinetics is to analyze the relationship between the change in mass gain, or oxide thickness, and time at specified temperatures. For the isothermal oxidation, the mass gain of the oxide over time generally follows the parabolic law [41]:

(1) $$\Delta W=\frac{W}{S}=kt^{0.5}$$

where Δ*W* is the unit mass gain after *t* time, *W* is the mass change, *S* is the apparent surface area of the specimen, and *k* is the diffusion kinetic coefficient. According to Fick’s second law under unsteady-state conditions, the one-dimensional oxygen diffusion coefficient from the oxide surface to the matrix can be described by the Arrhenius equation:

(2) $$k=k_{0}\exp(-\frac{Q}{RT})$$

where *k*_0_ is the kinetic empirical constant, *Q* is the thermodynamic activation energy, *R* is the gas constant, and *T* is the absolute temperature. Ignoring the interface influence between the ZrSi coating and the Zr-4 substrate, the mass gain of the coated specimen is equal to the sum of the mass gain of the ZrSi coating and the mass gain of the Zr-4 substrate. According to Equation (1), the mass gain of the ZrSi coating during oxidation can be obtained, as shown in Figure 11a. Then, the relationships between *k* and *T* during oxidation for the specimens are presented in Figure 11b. At the same temperature, the oxygen diffusion coefficient in the coating oxide is significantly smaller than that in the oxide of the Zr-4 substrate. Some of the oxidation thermodynamic parameters obtained in this study and those from previous studies are listed in Table 2. The value of *Q* for the Zr-4 substrate in this work approximates the results in the previous literature. The oxidation activation energy of the ZrSi coating is comparable to that of Si.

### 4.2. Oxide Analysis

**Figure 12:**
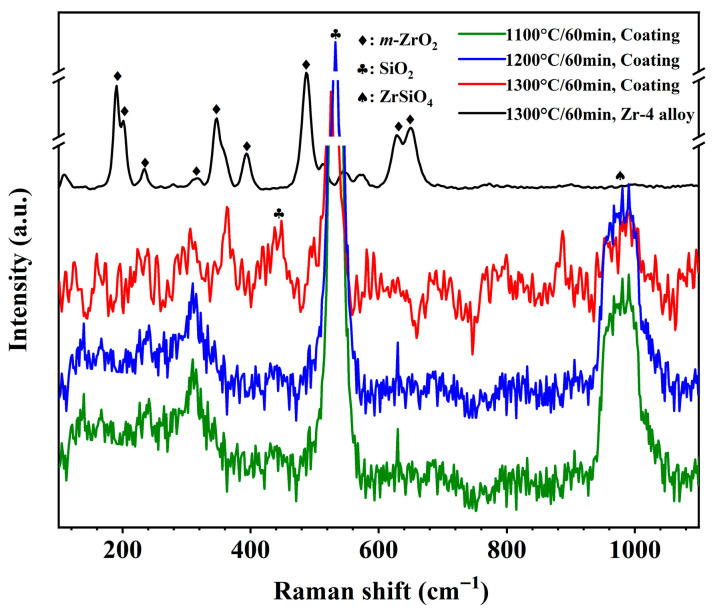
Raman spectra of the ZrSi coating and Zr-4 alloy oxidized under different conditions.

From the XRD analysis results in Figure 3, the oxides formed from laser cladding ZrSi coating are mainly ZrO_2_, ZrSiO_4_, and SiO_2_. After oxidation at 1100 °C for 20 min, the continuous oxide layer is not apparently detected on the ZrSi coating (Figure 5), which implies that the oxidation rate of the Si phase in the coating should be very low, leading to quite a thin oxide layer. To further study the oxide compositions on the coating surface, Raman spectra were used to analyze the compositions of the oxides, as shown in Figure 12. Raman shift at 998 cm^−1^ corresponds to the peak position of ZrSiO_4_ [45], the Raman shift at 520–550 cm^−1^ is the peak position of SiO_2_ [46], and the Raman shift at 330 cm^−1^ corresponds to the peak position of monoclinic ZrO_2_ [47]. These results coincide with the analysis in XRD tests. The oxide ZrSiO_4_ may be formed by the oxidation of ZrSi_2_, as shown in Equation (3):

2ZrSi_2_ + 6O_2_ → ZrSiO_4_ + ZrO_2_ + 3SiO_2_ (3)

**Figure 13:**
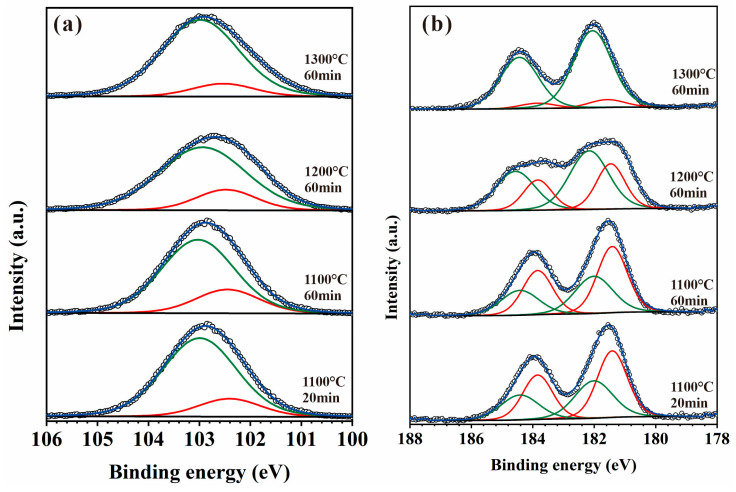
XPS spectra of (**a**) Si 2p and (**b**) Zr 3d of the surface oxides for the oxidized ZrSi coating.

**Figure 14:**
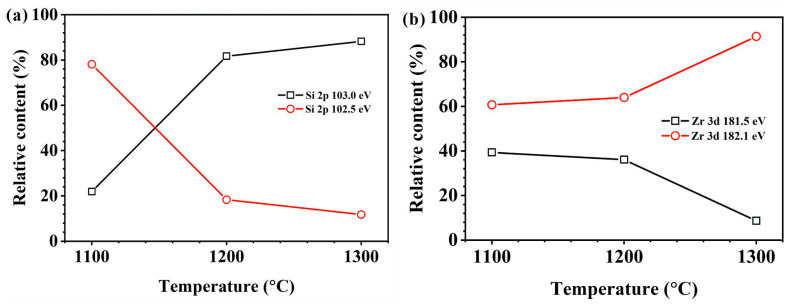
Relative content of the peak at (**a**) the Si 2p 103.0 eV and 102.5 eV, and (**b**) the Zr 3d 182.1 eV and 181.5 eV in the oxide surfaces after high-temperature oxidation for 60 min.

To further analyze the relative content of oxides after the ZrSi coating oxidation, XPS analysis was utilized, as shown in Figure 13. The peak binding energy at 103.0 eV corresponds to SiO_2_ [37], and there is also an obvious peak at 102.5 eV. In the crystal lattice of silica, each Si atom forms a tetrahedral coordination with four O atoms ([SiO_4_] unit), and with a Si-O bond length of ~1.61 Å and a Si-O bond energy as high as 452 kJ/mol [48], which is a typical strong covalent bond. In the lattice of ZrSiO_4_, Si is also in the [SiO_4_] tetrahedron. Due to the influence of the [ZrO_8_] polyhedron on the crystal structure, the Si-O bond length is approximately 1.62–1.64 Å; the bond is weakened by structural stress, thus the Si-O bond energy is slightly lower than that of silica, approximately 440–450 kJ/mol [49]. The electron binding energy of Si in ZrSiO_4_ is slightly lower than that of Si in SiO_2_. Therefore, the peak at 102.5 eV could be attributed to the peak of Si in ZrSiO_4_ (Figure 13a). The relative proportions for SiO_2_ and ZrSiO_4_ can be determined by analyzing the peak area ratios at 103.0 eV and 102.5 eV after oxidation for 60 min, as illustrated in Figure 14a. The content of SiO_2_ on the oxide surface gradually increases, while the ZrSiO_4_ content decreases with rising temperature. In Figure 13b, the peak value at 182.1 eV should be the peak of Zr^4+^ in ZrO_2_ [50]. Moreover, there is an obvious peak at 181.5 eV. In the lattice of monoclinic ZrO_2_, the Zr-O bond length ranges from 2.04 to 2.26 Å, and the bond length is ~2.1–2.2 Å in the lattice of cubic/tetragonal ZrO_2_ [51,52]. At room temperature, the Zr ion has a coordination number of seven in the monoclinic ZrO_2_, and an electronegativity difference (Δχ) that is 2.11 (Zr: 1.33, O: 3.44, Δχ = 2.11), indicating that the Zr-O bond has significant ionicity (~70% ionic bond component). For the ZrSiO_4_ affected by the Si-O covalent bond, Zr is in an 8-coordinate [ZrO_8_] triangular dodecahedron, connected with SiO_4_ tetrahedrons at corners. Although the electronegativity difference in ZrSiO_4_ is the same as that in ZrO_2_ (Δχ = 2.11), the presence of Si-O covalent bonds reduces the charge concentration of Zr, and results in slightly lower ionicity of the Zr-O bond, with a Zr-O bond length range of 2.13-2.29 Å (slightly longer than that in ZrO_2_) and moderately weaker bond strength [53]. As the ionicity of the Zr-O bond decreases, the electron binding energy of the Zr 3d orbital should decrease. Therefore, the peak at 181.5 eV in Figure 13b can be attributed to the peak of the Zr element in ZrSiO_4_. Based on the area ratio of different peaks, the relative content ratios of ZrO_2_ and ZrSiO_4_ can be obtained (Figure 14b). During oxidation for 60 min, the content of ZrSiO_4_ in the oxide decreases with the increase in temperature, coinciding with the results from Figure 14a. The Gibbs free energy of ZrSiO_4_ is lower than that of ZrO_2_ and SiO_2_. At relatively low temperatures, ZrSiO_4_ has a higher thermodynamic stability, and the oxidation of ZrSi_2_ tends to form ZrSiO_4_, which is consistent with the results on ZrSi oxidation reported by Hwasung Yeom [37]. As the temperature rises, the Gibbs free energies for ZrO_2_ or SiO_2_ formation also decrease, making it easier for ZrSi_2_ oxidation to form ZrO_2_ and SiO_2_:

ZrSi_2_ + 3O_2_ → ZrO_2_ + 2SiO_2_ (4)

It has been reported that during the initial oxidation of ZrSi_2_ at low temperature, unstable Zr-Si-O phases may be generated, and the unstable Zr-Si-O phase may decompose into stable SiO_2_ and ZrO_2_ with the increase in temperature and time [54]. The high-temperature oxidation of ZrSi_2_ is a solid-phase diffusion reaction, and the temperature required to form a stable ZrSiO_4_ phase needs to be higher than 1300 °C [55]. In this study, a large number of ZrSi_2_ laths are generated through rapid reactions in the liquid molten pool by a high-energy laser, which is different from the surface activity and stoichiometric ratio of the micro/nano ZrSi_2_ powders prepared by the solid-phase sintering methods [37,55,56]. This could be the reason for the differences in the oxidation behavior of ZrSi_2_ between this study and the previous results.

### 4.3. Microstructure of Oxide

The oxide surfaces exhibit different micro-morphologies after oxidation under different conditions in Figure 9. After exposure at 1100–1200 °C, the granular oxides with a size of ~1 μm were mainly formed by adhering to the ZrSi_2_ phase, and the surface roughness, which correlates to a certain degree with the oxide swelling height measured on the coating surface, is the greatest after oxidation at 1200 °C for 60 min (Figure 10). Combined with the results in Figure 9, it is proposed that the Zr and Si elements in the ZrSi_2_ phase migrate outward, resulting in the formation of granular oxides on the surface. Considering the volume swelling of the ZrSi_2_ phase after forming oxides, its volume change can be evaluated according to the Pilling–Bedworth Ratio (*PBR*), which can be used to predict whether the oxide layer has a protective effect [57]:

(5) PBR=VOxideVCompound≈∑MOxideερOxideMCompoundρCompound

where *V*, *M*, and *ρ* refer to molar volume, molar mass, and density, respectively, the subscripts “*Oxide*” and “*Compound*” denote the oxide and the metal compound, and *ε* represents the number of cations in the oxide. According to Equation (5), it can be estimated that the *PBR* value calculated using Equation (3) is 2.27, whereas the value obtained from Equation (4) is 1.6 following the oxidation of the ZrSi_2_ metal compound. When the *PBR* is >>1, the volume of the formed oxide is larger than that of the consumed material, and then the oxide undergoes considerable compressive stress, causing it to bend and crack, which leads to the formation of fractures that reveal new material surfaces vulnerable to further oxidation [58]. Thus, it can be inferred that irrespective of the formation of the ZrSiO_4_ phase following the oxidation of ZrSi_2_, the volume swelling of the oxide would grow considerably, potentially leading to compression and the development of fractures. This may be one of the reasons for the oxide cracking after oxidation at 1200 °C for 60 min (Figure 9d).

**Figure 15:**
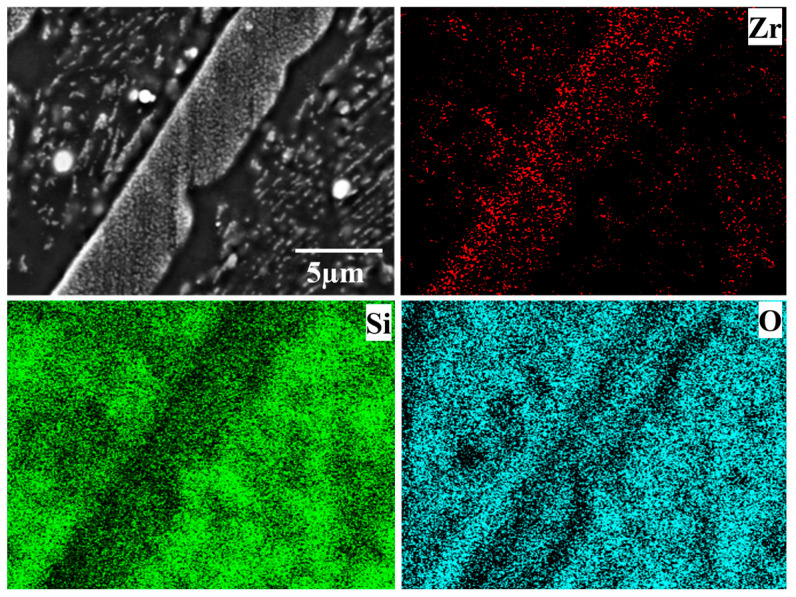
EDS mapping of the oxide on the coating surface at 1300 °C for 60 min.

After oxidation at 1300 °C, no obvious cracks appear in the oxide; however, the surface roughness of the oxide remaining stable over time is lower than that obtained at 1200 °C for 60 min. The formed SiO_2_ by the oxidation of ZrSi_2_ and Si is mainly a nano-sized amorphous phase [37]. The nano amorphous SiO_2_ may soften or even melt in a high-temperature environment, forming a typical glassy morphology [59], as shown in Figure 9e. The white nanoparticles in Figure 9f (with a size of 100–300 nm) should be mainly the ZrO_2_ phase formed by ZrSi_2_, which is wrapped by the glassy SiO_2_ phase, as shown in Figure 15. The cross-sectional oxide thickness of the large ZrSi_2_ laths after oxidation at 1300 °C is smaller than that formed at lower temperatures in Figure 6, which should be related to the formation of the amorphous SiO_2_ phase. In the study on the oxidation of MoSi_2_, it oxidizes to form a dense and continuous SiO_2_ glass film at temperatures above 900 °C, and these SiO_2_ phases uniformly cover the surface of MoSi_2_ [60]. The nano-sized zirconia uniformly dispersed in the SiO_2_ oxide film can enhance the oxidation resistance of the material [61]. The diffusion coefficient of oxygen in SiO_2_ is low, which can effectively isolate the contact between the matrix and oxygen, thus protecting the matrix. The viscosity of SiO_2_ is about 10^12^ Pa s at 1300 °C [62,63]. The viscosity of silicate melts is a complex function of temperature, melt chemical composition, and pressure. The temperature and composition strongly affect viscosity, leading to variations in several orders of magnitude. The contents of Fe and Sn in Zr-4 alloy are, respectively, 0.03 wt% and 1.5 wt%, and thus Fe and Sn are inevitably incorporated into the ZrSi coating during laser cladding and subsequently transferred into the oxide. The melting point of Fe_2_SiO_4_ is 1200 °C, and the oxide of Sn even has a lower melting point, 1127 °C. The two oxides that act as impurities to enter the glassy SiO_2_ can decrease the viscosity of the glass phase, eventually enabling a self-healing ability for glassy SiO_2_. The viscous SiO_2_ layer formed on the ZrSi coating at 1300 °C can also act as a healing agent to greatly relieve the growth stress between the bulging oxide generated by the large ZrSi_2_ lath and the surrounding oxide from the Si phase and reduce the oxidation of the coating (Figure 9e). According to the results in Figure 10, the surface roughness of the oxide layer gradually increases with increasing oxidation time at 1200 °C. Combined with the observations in Figure 9c,e, it can be concluded that the geometric size of oxide particles on the surface increases progressively, leading to an increase in roughness. At 1300 °C, the diffusion rates of O and Si become faster based on the principle of oxidation kinetics, and the growth rate of oxides on the coating surface should increase, consequently, resulting in greater surface roughness. However, contrary to this prediction, the surface roughness of the oxide obtained at 1300 °C for 60 min was lower than that measured under 1200 °C/60 min. This issue should be associated with the softening of the SiO_2_ oxide formed on the coating surface. Due to the effect of surface tension, the softened SiO_2_ forms a continuous oxide film despite its relatively high viscosity, reducing the geometric size of surface oxides and thereby decreasing the surface roughness. The viscous SiO_2_ film is more sensitive to temperature variations, which enable the surface roughness of the oxide to remain stable as the oxidation time increases. At 1100 °C and 1200 °C, due to the large oxide growth stress after ZrSi_2_ oxidation, cracks are generated in the oxide. The SiO_2_ layer that remains solid at lower temperatures cannot prevent the oxide cracks from healing, resulting in significant oxidation of the large ZrSi_2_ laths (Figure 4a–f).

According to the results in Figure 2 and Figure 11b, it can be seen that the oxidation mass gain and oxidation activation energy of the ZrSi coating are both lower than those of the Zr-4 alloy. For the oxidation behaviors of Cr [64] and CrAl [65] coatings, their oxide thicknesses were, respectively, ~20 µm and ~22 µm at 1200 °C for 60 min in steam, which were comparable with the oxide thickness from the large ZrSi_2_ lath (Figure 6); the internal oxidation occurred for the Zr alloy coated by Cr and CrAl layers; however, the ZrSi coating was free from this issue, and the oxide thickness of the ZrSi coating decreased at higher temperature. The oxidation activation energy for the Ti_2_AlC MAX phase was 175 kJ/mol during oxidation at 200–1000 °C [66], which was a little lower than that of the ZrSi coating. The laser cladding ZrSi coating can provide oxidation protection for the Zr-4 alloy under high-temperature conditions. At temperatures below 1200 °C, the oxide film of the large lath-like ZrSi_2_ is prone to crack under growth stress, leading to further oxidation of the ZrSi_2_ phase. At 1300 °C, the glassy SiO_2_ formed by Si and ZrSi_2_ phases exhibits some viscosity, enabling the oxide layer’s self-healing capabilities, which can inhibit the inward diffusion of oxygen, thus providing high-temperature protection for the ZrSi coating.

## 5. Conclusions

To enhance the high-temperature oxidation resistance of Zr-4 alloy, this study deposited Si powder on the surface of Zr-4 alloy using laser cladding. Through rapid heating and convection in the molten pool, a ZrSi coating consisting of ZrSi_2_ and Si was formed. The oxide evolution of the ZrSi coating was studied under high-temperature water–steam conditions, and the following conclusions were drawn:

(1) During the laser cladding processes, Si and Zr alloy reacted to form the ZrSi_2_ phase, and a large number of ZrSi_2_ laths grew along the cooling gradient. The laser cladding ZrSi coating comprised the ZrSi_2_ phase and the Si-rich phase.

(2) The weight gain curves for the ZrSi coating and Zr-4 alloy followed the parabolic law when subjected to water–steam environments at temperatures between 1100 °C and 1300 °C. The oxidation thermodynamic activation energies of the ZrSi coating and Zr-4 alloy were 182.7 kJ/mol and 82.5 kJ/mol, respectively.

(3) The ZrSi_2_ phase predominantly generated ZrSiO_4_, ZrO_2_, and SiO_2_ oxides after high-temperature exposure, as determined by the analysis, e.g., Raman and XPS tests. After oxidation at 1100 °C for 60 min, due to the higher thermodynamic stability of ZrSiO_4_, its relative content formed by ZrSi_2_ oxidation was higher than that of ZrO_2_; however, it gradually decreased as the temperature rose.

(4) During heat exposure, oxygen diffused into the ZrSi coating, and the Si and Zr in the ZrSi_2_ phase diffused outward, forming granular oxides on the coating surface. Compared with the Si-rich phase, a larger growth stress occurred in the oxides formed by ZrSi_2_, which can cause cracks in the oxides during exposure at 1100 °C and 1200 °C.

(5) At 1300 °C, the amorphous viscous SiO_2_ formed by the ZrSi_2_ and Si-rich phases can heal the cracks within the oxide of ZrSi_2_, and its low oxygen diffusion coefficient endows protection for the underlying matrix.

## Figures and Tables

**Table 1:** The element composition (at. %) of the area marked in Figure 1c,e.

Element	Zr	Si	O
A	32.47	65.12	2.41
B	86.89	12.34	0.77
C	100	-	-
D	31.81	65.42	2.77
E	1.51	96.01	2.48

**Table 2:** Relevant parameters of material oxidation in this study and some previous research works.

Material	Temperature (°C)	*k*_0_ (gm^−2^s^−0.5^)	*Q* (kJ/mol)
Zr-4	1100–1300	71	82.5
ZrSi coating (laser cladding)	1100–1300	44,241	182.7
ZIRLO^TM^ [41]	1000–1400	16,079	82.8
Zry-4, steam [42]	1000–1600	6020	83.6
Zry-4 [43]	1050–1400	2980	75.1
Si [44]	800–1200	/	154.4–212.6

## Data Availability

The original contributions presented in this study are included in the article. Further inquiries can be directed to the corresponding authors.
